# Structure based modification of Bluetongue virus helicase protein VP6 to produce a viable VP6-truncated BTV

**DOI:** 10.1016/j.bbrc.2014.08.028

**Published:** 2014-09-05

**Authors:** Eiko Matsuo, Esther Leon, Steve J. Matthews, Polly Roy

**Affiliations:** a Microbiology & Immunology, Division of Animal Science, Department of Bioresource Science, Graduate School of Agricultural Science, Kobe University, 1-1, Rokkodai, Nada-ku, Kobe-City 657-8501, Japan; b Faculty of Infectious and Tropical Diseases, London School of Hygiene and Tropical Medicine, Keppel Street, London WC1E 7HT, UK; c Division of Molecular Biosciences, Centre for Structural Biology, Imperial College London, South Kensington, London SW7 2AZ, UK

**Keywords:** Bluetongue virus, VP6, Multinuclear nuclear magnetic resonance, Reverse genetics

## Abstract

Bluetongue virus core protein VP6 is an ATP hydrolysis dependent RNA helicase. However, despite much study, the precise role of VP6 within the viral capsid and its structure remain unclear. To investigate the requirement of VP6 in BTV replication, we initiated a structural and biological study. Multinuclear nuclear magnetic resonance spectra were assigned on his-tagged full-length VP6 (329 amino acid residues) as well as several truncated VP6 variants. The analysis revealed a large structured domain with two large loop regions that exhibit significant conformational exchange. One of the loops (amino acid position 34–130) could be removed without affecting the overall fold of the protein. Moreover, using a BTV reverse genetics system, it was possible to demonstrate that the VP6-truncated BTV was viable in BHK cells in the absence of any helper VP6 protein, suggesting that a large portion of this loop region is not absolutely required for BTV replication.

## Highlights

• NMR analysis on BTV VP6 reveals two large loop regions.
• The loss of a loop (aa 34–130) does not affect the overall fold of the protein.
• A region of VP6 (aa 34–92) is not required for BTV replication.
• A region of VP6 (aa 93–130) plays an essential role in the virus replication.

## 1 Introduction

Bluetongue virus (BTV), the etiological agent of Bluetongue disease of livestock, is a member of the *Orbivirus* genus of the *Reoviridae* family. BTV particles have three consecutive proteins layers that are organized into two capsids; an outer capsid comprising VP2 and VP5 and an inner icosahedral capsid (core) composed of two major proteins, VP7 and VP3, which encloses the three minor proteins, VP1, VP4 and VP6, in addition to the viral genome. The viral genome consists of 10 linear dsRNA molecules, segment 1 to segment 10 (S1-S10). In addition to 7 structural proteins, BTV genome also encodes 3 or 4 nonstructural proteins (NS1, NS2, NS3 and NS4) in infected host cells [1], [2], [3].

The catalytic activities of both VP1 and VP4 have been confirmed by a range of *in vitro* studies [4], [5], [6], [7], [8]. Furthermore, structural studies have revealed their close association in a complex located at the 5-fold vertices of the VP3 subcore [9], [10]. In contrast, despite considerable information regarding its enzymatic function of VP6 *in vitro* [11], [12], little is known regarding its structure or location in the BTV core. While VP6, like VP1 and VP4, is co-purified with core from virus-infected cells, VP6 is not readily taken up into core-like particles (CLPs) when co-expressed with major core structural proteins VP3 and VP7 [10], [13]. Furthermore, although VP6 is likely to unwind dsRNA either ahead or behind the transcribing polymerase [11], [12], it is also possible that the helicase activity could play an entirely different role in virus assembly.

Recently, we constructed VP6-deficient BTV strains using a reverse genetics (RG) system and a complementary BSR-VP6 cell line, demonstrating that VP6-deficient mutant viruses were replication-deficient in non-complementary cells (normal BSR cells) [14], [15]. Moreover, we demonstrated that the BTV core particle, purified from normal BSR cells infected with VP6-deficient mutant virus, contained neither the genomic dsRNA nor the two proteins of polymerase complex, VP1 and VP4 [16]. Thus, it is reasonable to hypothesize that VP6 plays a role in RNA packaging in addition to its role as a helicase in BTV mRNA transcription [16].

In this study, to further understand the importance of VP6, we assigned Multinuclear nuclear magnetic resonance (NMR) spectra of VP6 to provide insight into the VP6 structure. Based on NMR-derived secondary structure, it was possible to design a series of truncated proteins removing dynamic loop regions. Most importantly, in spite of the essential role of VP6 in the primary replication cycle of BTV, we show that the region between aa 34 and aa 92 of this large loop is not required for viral replication. However the second half of this loop (aa 93–130) plays an essential role in virus replication.

## 2 Materials and methods

### 2.1 Cell lines

BSR cells (BHK-21 subclone) were maintained in Dulbecco’s modified Eagle’s medium (DMEM) (Sigma) supplemented with 4% (vol/vol) fetal bovine serum (FBS) (Life Technologies). The stable cell line, BSR-VP6 [15], were grown in DMEM-4% FBS supplemented with 7.5 μg/ml of puromycin (Sigma).

Preparation of dsRNA was as described previously [17], [18], [19], [20].

### 2.2 Plasmids and BTV T7 transcripts

A bacterial expression plasmid for the fusion of BTV-10 VP6 protein incorporating N-terminal hexahistidine tag (his-tag) was produced by inserting the coding region of S9 into the modified bacteria expression vector, pRSETA-imperial. A region, which includes transcript stabilizing sequence from gene 10 of phage T7, the Xpress™ epitope, and the enterokinase cleavage recognition sequence, was replaced with the thrombin cleavage recognition sequence.

Mammalian expression plasmids for the RG system, pCAG-PBTV1VP1, pCAG-PBTV1VP3, pCAG-PBTV1VP4, pCAG-PBTV10VP6, pCAG-PBTV1VP7 and pCAG-PBTV1NS2, were as described previously [15], [16]. T7 plasmids for BTV transcripts used in the RG system were as described previously [15], [18].

Modification of VP6 and S9 was generated by site-directed mutagenesis, using the method as described previously [21]. The sequence of each modified VP6 and S9 plasmid was confirmed.

Synthesis of uncapped BTV transcripts was performed as described previously [15]. For synthesis of uncapped T7 transcripts, RiboMAX Large Scale RNA Production System-T7 (Promega) was used according to the manufacturer’s protocols.

### 2.3 Expression and purification of a series of BTV VP6 proteins in an *Escherichia coli* strain

An *E. coli strain*, BL21, was transformed with each VP6 expression plasmid. The transformed cells were incubated in 10 ml of LB broth containing 100 μg/ml of ampicillin (ABPC) at 37 °C for 16 h. After incubation, 1 ml of culture was inoculated to 10 ml of the fresh LB broth containing 100 μg/ml of ABPC and was incubated at 37 °C for 1 h. The expression of his-tagged VP6 was then induced for 5 h with the presence of 0.5 mM of IPTG.

For the production of labelled NMR, sample BL21 (DE3) was used. After the transformation, a clone for each protein was selected and amplified in 1 L of LB broth upon reaching OD_600_ ∼ 0.3–0.5. The cell pellets were transferred from the culture to 1 L of minimal media containing ^15^NH_4_Cl and ^13^C_6_-glucose in D_2_O for efficient ^15^N-^13^C-labelling and deuteration of VP6. Expression of the his-tagged protein was induced after 5 h by adding 0.5 mM IPTG, and then harvested after an overnight induction period at 28 °C. Note that for the expression of mutated VP6 proteins, an optimized expression protocol based on the double-colony selection method described previously was used [22].

The cells were collected by centrifugation and lysed by sonication. The his-tagged protein was purified by bench top chromatography using a nickel–nitrilotriacetic acid (Ni–NTA) resin. A second step of size exclusion chromatography was carried out on the previous protein enriched fractions. Protein samples were concentrated to 0.75 mM in 50 mM sodium phosphate pH7.5, 50 mM NaCl, 50 mM l-arginine, 50 mM l-glutamic acid, 10 mM DTT and 10% D_2_O.

### 2.4 Acquisition of NMR spectra

NMR spectra were collected at 298 K on Bruker DRX600 and DRX800 spectrometers equipped with Z-shielded gradient triple resonance cryoprobes. The chemical shifts of ^1^HN, ^15^N, ^13^C_α_, ^13^C_β_ and ^13^CO cross peaks were assigned using the double- and triple-resonance heteronuclear three-dimensional NMR spectra and in-house assignment algorithms [23].

### 2.5 Transfection of cells

Confluent monolayers of BSR-VP6 cells were transfected, first with six mammalian expression plasmids (100 ng each), coding VP1, VP3, VP4, VP6, VP7 and NS2, followed by the second transfection with ten BTV T7 transcripts (50 ng each) using Lipofectamine 2000 Reagent (Life technologies) as described previously [16]. At 6 h post second transfection, the culture medium was replaced with fresh DMEM containing 5% FBS and the plates were incubated at 35 °C in 5% CO_2_ for 3 days to allow cytopathic effects (CPE) to appear. Note that as the recovery efficiency should be low due to uncapped transcription in the second transfection, the plaque-purification procedure was not performed after transfection in this study.

### 2.6 BTV replication assay

To observe CPE, 10 μl of VP6-truncated BTV recovered from transfected BSR-VP6 cells was inoculated to normal BSR cells and the supernatant, containing viruses, was replaced with 1.5% low-melting agar in D-MEM supplemented with 5% FBS. At 2 days post-inoculation, cells were stained with 0.5% of crystal violet.

To determine the virus replications, each 100 μl of VP6-truncated BTV recovered from transfected BSR-VP6 cells were once amplified in BSR-VP6 cells (P0), followed by the second (P1) and the third (P2) amplification in normal BSR cells. The virus titer of each collected supernatant (P0 ∼ P2) was determined by plaque assay using BSR-VP6 cells.

## 3 Results

### 3.1 Expression of VP6 in *E. coli* and structural characterization by NMR

**Fig. 1:**
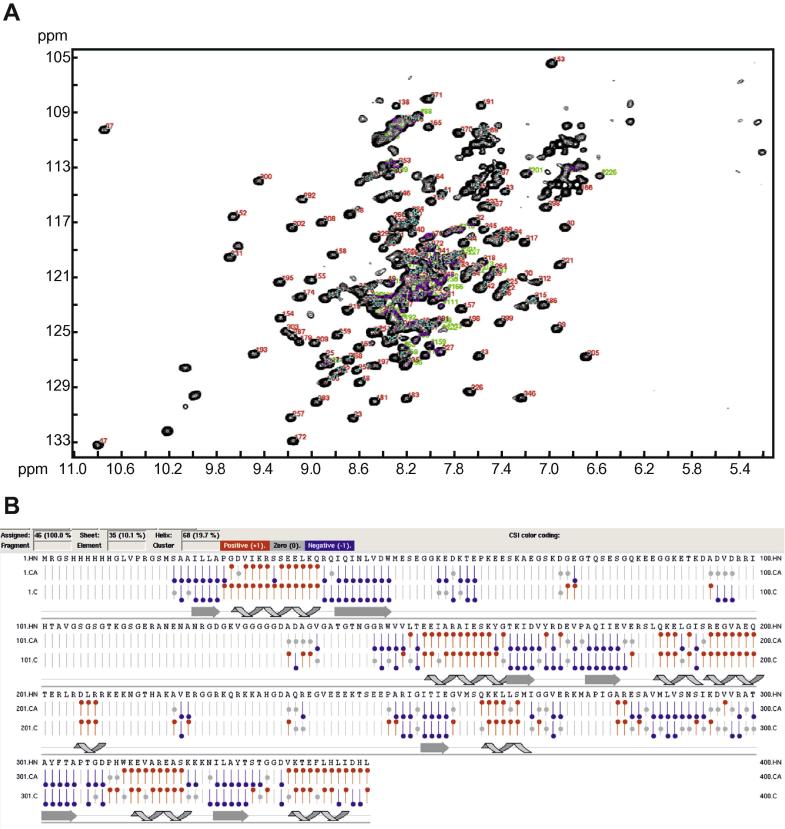
The sequence specific assignment of the ^1^H-^15^N-HSQC NMR spectrum of the ^15^N, ^13^C-labelled VP6. (A) Assignment on ^15^N-HSQC spectrum of 0.75 mM VP6 full-length. (B) The CSI plot for the Cα, and CO atoms. The CSI plot of native VP6 carbon atoms is based on the sequential resonance assignment of the triply labeled protein mentioned above. The CSI plot was generated using NMRview [26] and is shown below the amino acid sequences of the his-tagged VP6. The predicted secondary structure was shown under the plot. The positions of ß-strands and α-helix, which were calculated from the atomic coordinates, were indicated as gray arrows and looped lines, respectively.

To facilitate the NMR study, BTV-10 VP6 was overexpressed with an N-terminal his-tag in *E. coli*. The proteins were expressed well in soluble form (data not shown). The NMR analysis was first performed on the full-length VP6 (329 residues). Sequence specific assignment of NMR spectra of ^15^N, ^13^C-labelled VP6 could be completed for over half the protein sequence (Fig. 1A), whilst the remaining sequence formed two large dynamic loops. Conformational exchange on intermediate timescale within these regions broadened the majority of the NMR signals beyond detection. As shown in Fig. 1B, the chemical shift index (CSI) plot for backbone atoms of the full-length VP6 was obtained based on the sequential resonance assignment of the triple-labeled protein. There were two gaps in the assigned sequence (from aa 33 to aa 130 and aa 184 to aa 231), which likely indicate the presence of dynamic loops that exhibit conformation exchange, as the corresponding cross-peaks did not appear in the spectra.

### 3.2 Design of the truncated BTV VP6 variants

**Fig. 2:**
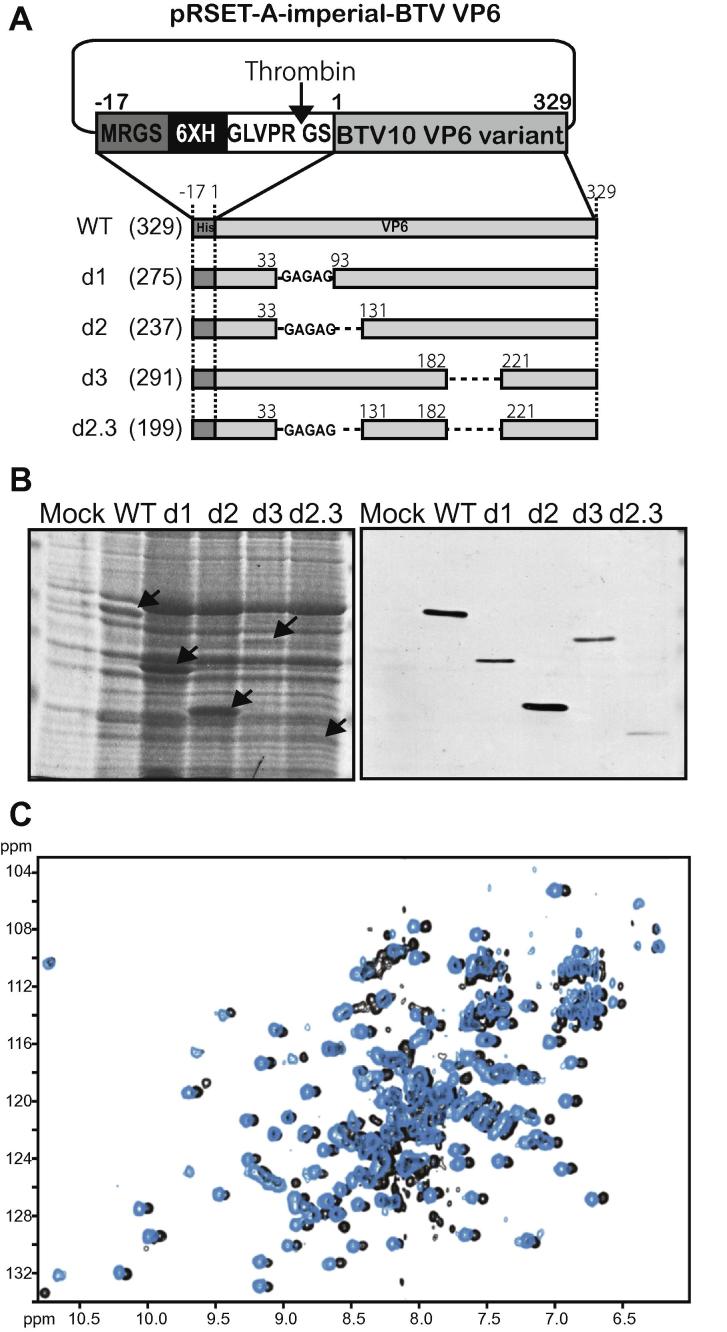
Construction of the truncated VP6 variants. (A) Schematic representation of the changes introduced in the his-tagged VP6 of BTV. Either or both of two “invisible” regions were truncated. On the left, the name of mutation is indicated. Numbers indicate amino acid positions in VP6 where deletions were introduced. To keep flexibility, the amino acid sequence, GAGAG, was inserted into a truncated region of the first loop. Total length of each protein is indicated. (B) Each VP6 variant was expressed in *E. coli* strain BL21 and confirmed by SDS–PAGE (left panel) and immunoblotting analysis using anti-polyhistidine antibody (right panel). (C) Comparison of ^15^N, ^13^C-d2VP6 spectrum (black) with ^15^N, ^13^C-WTVP6 spectrum (cyan). Note that the cyan spectrum is shifted in the 1H dimension for clarity to illustrate the similarity better. (For interpretation of the references to color in this figure legend, the reader is referred to the web version of this article.)

The presence of the two dynamic loops poses a significant challenge for the high-resolution structural study of VP6, especially as their signals are undetectable in NMR spectra and the conformational plasticity likely prevents crystallization. We therefore designed several shorter constructs of the protein VP6 based on our NMR assignment that either removed one or both loops (Fig. 2A).

The four deletions were created in his-tagged VP6 protein. The mutants of VP6, d1 and d2, lacked the first loop, either a portion (aa 34–aa 92; d1) only or the complete loop region (aa 34–aa 130, d2) of VP6. The third truncated VP6, d3, had a deletion of the second loop region (aa 183–aa 220) and the last mutant VP6, d2.3, lacked both loop regions (aa 34–aa 130 and aa 183–aa 220). To reduce any detrimental effects on the stability of the core VP6 domain, we substituted the first loop in variants d1, d2 and d2.3 with 5 amino acid residues, Gly-Ala-Gly-Ala-Gly (GAGAG). All truncated VP6 proteins were expressed in *E. coli* strain, BL21 (Fig. 2B). Truncations of the loop between residues 183 and 220 rendered constructs unstable, and the expressed protein were either insoluble, as for d2.3VP6 construct, or not expressed at a detectable level, as for d3VP6. However, deletion of the first loop (residues 34–130) did not affect expression yields or the protein structure as tested by preliminary NMR spectra (data not shown). Therefore, labeled samples were produced for construct d1VP6 and d2VP6, following the protocol described previously for full-length VP6 (WTVP6).

Protein samples were concentrated to 0.75 mM in 50 mM sodium phosphate pH7.5, 50 mM NaCl, 50 mM l-arginine, 50 mM l-glutamic acid, 10 mM DTT and 10% D2O. By simple comparison of the sequence specific assignment of WTVP6 on the ^1^H-^15^N-HSQC spectrum shown in Fig. 1A, many of the signals could be assigned unambiguously for the short deletion construct (d1VP6, data not shown), demonstrating that the lack of this major loop did not affect the structure of core domain, even when the entire loop was removed (d2VP6, data not shown). NMR studies were continued using the construct d2VP6 since it is shorter than d1VP6, and the expressed protein product was at a much higher level than that of d1VP6 after purification.

A ^15^N, ^13^C-double labeled sample of d2VP6 was expressed and purified as described above. The sequence assignment of ^15^N-^13^C-d2VP6 on the ^1^H-^15^N-HSQC spectrum was very similar to that of WTVP6, suggesting that loss of the loop did not affect the overall folding of the protein (Fig. 2C). The assignment of the sequence was almost complete, with the exception of the residues from the flexible loop towards the end of the sequence, which were not visible in the spectra. Although it was not possible to perform three-dimensional structural calculations with this limited data, it is clear that significant portions of VP6 are not necessary for keeping the structure in stable form.

### 3.3 VP6-truncated virus d1BTV replicates in normal cells but d2BTV does not

**Fig. 3:**
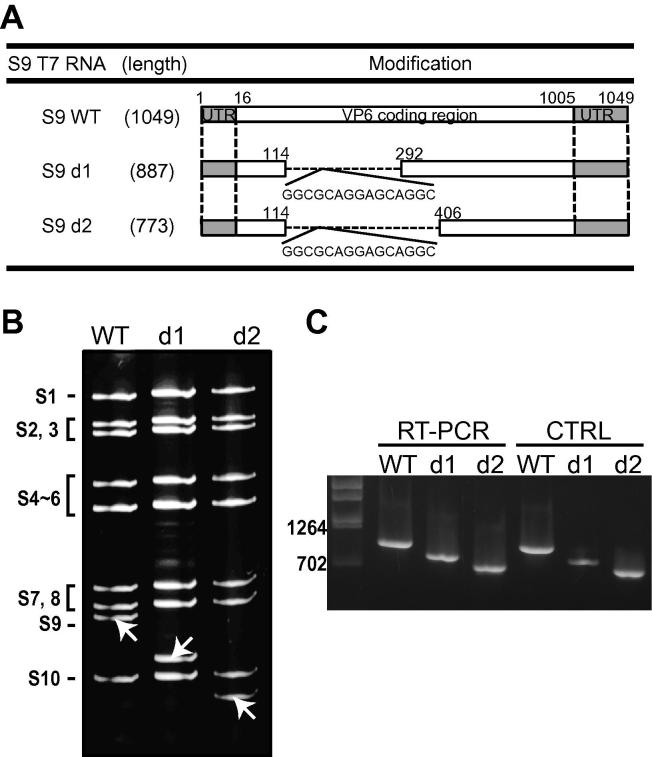
Recovery of VP6-truncated BTV from complementing cell line BSR-VP6. (A) Schematic representation of the changes introduced in the segment S9 of BTV. On the left, the name of mutation is indicated. Numbers indicate nucleotide positions in the S9 where deletions were introduced. The nucleotide sequence coding 5 amino acid residues, GAGAG, was inserted as indicated. Total length of each segment is indicated. (B) Genomic dsRNA was purified from infected cells and analyzed by nondenaturing-PAGE. (C) cDNA copies of S9 were synthesized from isolated viral dsRNA and amplified by PCR (lanes 2–4). As a control, PCR products were amplified from T7 plasmids of S9 variants (lanes 5–7). Markers (lane 1) are indicated. The numbers on the left indicate the markers nucleotides.

NMR study revealed that two truncated VP6 mutants, d1 and d2, possessed similar structures to full-length VP6 (WTVP6). To determine whether these truncated VP6 proteins were still functional in BTV replication, the two VP6-truncated viruses, which contained the same deletions in S9 RNA segment as those in his-tagged VP6, were generated (Fig. 3). The two mutations designed in S9 included either a half-deletion of the first flexible loop of VP6 (nt 115–nt 291) (d1), or a whole deletion (nt 115–nt 405) (d2) (Fig. 3A). The deleted regions in both d1 and d2 were substituted with the 15 mer-sequence coding 5 residues, GAGAG, in frame.

Each virus was recovered in the BSR-VP6 complementing cell line and the profile of the genomic dsRNAs of each mutant virus was analyzed by native-PAGE (Fig. 3B). The presence of the designed truncation/substitution in each mutated virus was confirmed by sequencing the corresponding RT-PCR product (Fig. 3C and not shown). No unexpected changes were detected, indicating that all mutant segments were functional in genome packaging and replication in complementary BSR-VP6 cells.

**Fig. 4:**
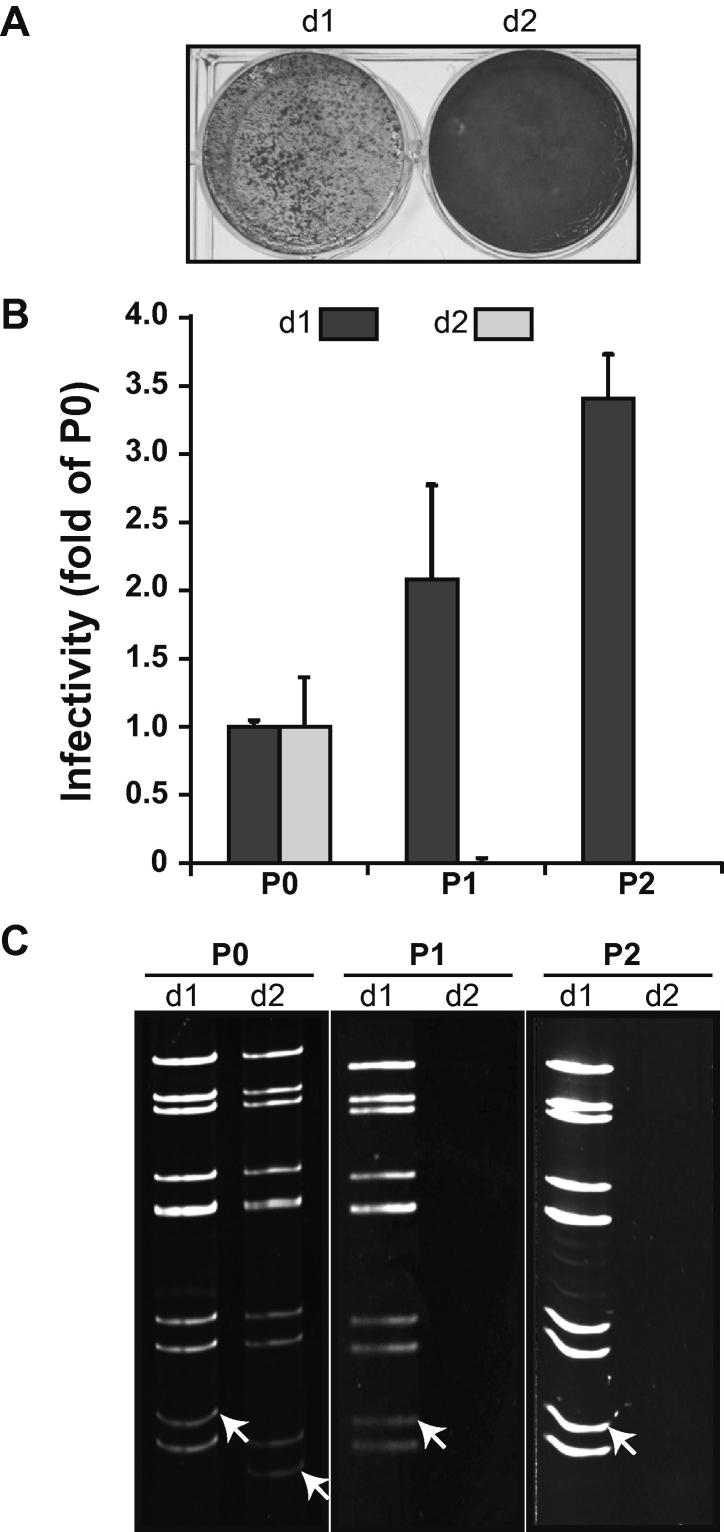
Characterization of VP6-truncated BTV in normal BSR cells. (A) Normal BSR cells infected with 10 μl of VP6-truncated BTV were stained with 0.5% crystal violet at 2 days post-infection. (B) Each 100 μl of VP6-truncated BTV recovered from transfected BSR-VP6 cells were once amplified in BSR-VP6 cells (P0), followed by the second (P1) and the third (P2) amplification in normal BSR cells. The virus titer of each collected supernatant (P0 ∼ P2) was determined by plaque assay using BSR-VP6 cells and the infectivity was shown as a fold of P0 (Mean ± SD). (C) Pattern of genomic dsRNA purified from cells infected with d1BTV and d2BTV (P0 ∼ P2) was analyzed by nondenaturing-PAGE. Position of the corresponding S9 in each VP6-truncated virus is indicated with white arrows.

To assess the replication capability of the VP6 mutant viruses in normal BSR cells, 10 μl of VP6-truncated BTV recovered from transfected BSR-VP6 cells was inoculated into normal BSR cells. At 2 days post-inoculation, the presence of CPE was observed (Fig. 4A). Surprisingly, the inoculation of d1BTV induced pronounced CPE to the normal BSR cells whereas no CPE was observed in d2BTV-infected BSR. Note that this is the first report of a viable VP6-truncated BTV in normal BSR without any helper VP6 protein.

To further confirm viability of d1BTV in normal BSR, the infectious virus titer (cell-free, supernatant virus) and the profile of genomic dsRNAs extracted from infected cells in each virus passage (P0 ∼ P2) were determined (Fig. 4B and C). The titer of d1BTV dramatically increased during passage using normal BSR (Fig. 4B, dark gray columns). Moreover, the dsRNA profile of d1BTV in each passage showed that the truncation was absolutely retained (Fig. 4C). The presence of the mutation in each passage was confirmed by sequencing the corresponding RT-PCR product (data not shown). In contrast, d2BTV could not replicate in normal BSR at all (Fig. 4B, light gray columns). In addition, the genomic dsRNAs of d2BTV were only detectable in BSR-VP6 cells (Fig. 4C, P0). These results clearly suggested that a part of VP6 (nt 115–nt 291; aa 34–92) was not required for BTV replication in BSR cells, whereas a latter half of the first flexible region may possess the unknown but essential function in the virus replication.

## 4 Discussion

BTV, a member of the *Orbivirus* genus, is unique among the *Reoviridae* family in encoding VP6, a protein with nucleoside triphosphatase, RNA binding, and helicase activity *in vitro* [11], [12], [24]. In addition, we recently reported that VP6 plays an essential role in primary replication and possibly in RNA packaging during core assembly as well [14], [15], [16]. Although it is reasonable to hypothesize that VP6 also plays a role in RNA packaging in addition to its role as a RNA helicase, the exact function of BTV VP6 in BTV biology still remains to be addressed, as to date it has not been possible to determine its structure or its precise location within the core. In this paper we have presented a preliminary NMR study on BTV VP6 to understand further its function in BTV replication. Using a combination of solution NMR spectroscopy, RG system and site-specific mutagenesis, we have demonstrated that a significant fragment of VP6 is not absolutely necessary for BTV replication, at least in cell culture.

Despite decades of attempts to generate an atomic structure of VP6, it has not been possible to crystallize the protein. VP6 protein is highly soluble and can only be precipitated in the presence of up to 90% (wt/wt) of the ammonium sulfate [12]. We therefore used NMR to understand the structural features of the protein in solutions. This data showed that half of the protein forms two large loop regions, whose conformational dynamics makes them undetectable by NMR as the signals are broadened. These dynamic regions in VP6 likely contribute to the high solubility of VP6, resulting in difficulties for crystallization. Since the truncation of the first large loop of VP6 retains the same core structure to full-length VP6, it is possible that this truncation may allow future crystallization of VP6.

One of the truncations, the complete truncation of the first loop (d2), lost the function of VP6 in BTV replication whereas the half truncation of that (d1) still retained the active function. The lack of 38 residues between amino acid position 93 and 130 in VP6 is likely to be critical in BTV replication. Previous *in vitro* studies showed that this region of d2VP6 contains ATP-binding/ATPase motif [11], suggesting that d2BTV could not replicate possibly due to loss of ATPase activity. However, the program ATPint, which predicts ATP-interacting residues, (http://www.imtech.res.in/raghava/atpint/), suggests that the 10 residues in d2VP6, which included the 5 linker residues inserted into the truncated loop, may interact with ATP. Therefore, it is conceivable that the insertion of the linker may have created an unexpected ATPase motif that allows d2VP6 to complement for VP6’s ATPase function during BTV replication.

Recently, the fourth nonstructural protein of BTV, NS4, was identified [1]. NS4 is encoded by an ORF (nt 182–415) in S9 overlapping the ORF encoding VP6. The deletion of NS4 by removing the initiation codon (nt 182 in S9) and the induction of two stop codons at amino acid position 24 and 67 of NS4 was dispensable for BTV replication [1]. However, this mutated S9 could still express a part of C-terminal region of NS4 translated from the initiation codon at position nt 323, originally coding methionine at amino acid position 48 in NS4 and terminating at the inducing stop codon. In our mutated S9, the deletion of a whole first flexible region (d2S9) resulted in the complete deletion of the NS4 protein. On the other hand, d1S9 still retained the ability to express 30 residues of C-terminal region of NS4 as ATG at position nt 161 in d1S9, which is matched with nt 323 of WTS9, and could work as initiation codon with medium Kozak context, gAgaATGc [25]. Thus, although further studies are necessary, it is still possible that C-terminal of NS4 may have an important role in BTV replication.

The deletion of VP6 was believed to be replication-defective as VP6 protein is essential for the primary replication [14], [15], [16]. However, here we first demonstrated that the truncation of 59 residues in VP6 (177 nt of S9) did not affect BTV replication. Moreover, the deletion was stable over passage of d1BTV in mammalian cells. Further study is required to clarify whether the truncated region is definitely unnecessary for BTV replication in both insect cells and mammalian cells as well as in natural hosts.
